# Exploring the Relation Between Peripapillary Retinal Vessel Caliber and Visual Field Defects in Primary Open-Angle Glaucoma

**DOI:** 10.7759/cureus.71538

**Published:** 2024-10-15

**Authors:** Rekha R Mudhol, Arkaprava Ray

**Affiliations:** 1 Ophthalmology, Shri Basanagouda Mallanagouda (BM) Patil Medical College, Bijapur Lingayat District Educational University (BLDE) (Deemed to be University), Vijayapura, IND

**Keywords:** fundus photo, glaucoma practice, open-angle glaucoma, primary open-angle glaucoma, visual field defect

## Abstract

Background

Primary open-angle glaucoma (POAG) is a progressive optic neuropathy characterized by loss of retinal ganglion cells, optic nerve head changes, and visual field defects. Vascular factors, alongside intraocular pressure, play a crucial role in POAG pathogenesis, and this study aims to explore the relationship between retinal vessel caliber and visual field defects in POAG patients.

Purpose

To evaluate the association between retinal vessel calibers in the peripapillary area with visual field defects in one or both hemifields.

Materials and Methods

This cross-sectional study was conducted at a tertiary eye care center in northern Karnataka for one year, enrolling 100 eyes from 50 primary open-angle glaucoma (POAG) patients with visual field abnormalities. Fundus color stereoscopic photographs were taken using a Canon digital camera, and diameters of the superior and inferior temporal arteries (STA and ITA) and veins (STV and ITV) in the peripapillary area were measured. Visual field defects were categorized, and statistical analyses were performed to correlate retinal vessel calibers with field defect locations.

Results

Participants had a mean age of 89.68 years, with 72% male. Visual field defects included superior arcuate scotoma (20%), inferior arcuate scotoma (32%), double arcuate scotoma (30%), and tubular vision (18%). Significant vessel caliber changes were observed: for superior hemifield defects, the mean STA: ITA ratio was 1.15 (p = 0.001), and for inferior defects, it was 0.94 (p = 0.04). STV: ITV ratios also showed significant changes corresponding to visual field defects. Eyes with defects in both hemifields showed pronounced narrowing, particularly in inferotemporal vessels.

Conclusions

Decreased retinal vessel caliber is significantly associated with visual field defects in primary open-angle glaucoma and can be a valuable diagnostic parameter tool for assessing glaucomatous damage and the progression and severity of POAG.

## Introduction

Primary open-angle glaucoma (POAG) is a progressive loss of retinal ganglion cells and axons, leading to characteristic changes in the optic nerve head with corresponding defects in the visual field [1]. The worldwide prevalence of POAG was 1.96% of the total general population in 2010, affecting more women and Asians [2]. Glaucoma is the second most common cause of blindness in Indian adults [3]. Although many risk factors have been found, it is still difficult to predict or identify the early progression of glaucoma since visual field defects are only noticeable when at least 40% of the retinal ganglion cell axons have been lost [4,5].

Elevated intraocular pressure (IOP) is a key modifiable risk factor for glaucoma, and reducing IOP effectively decreases the risk and progression of the disease, supporting the mechanical theory of the pathogenesis of glaucoma [6]. Normal tension glaucoma (NTG), a subset of POAG, can occur even when IOP is within normal ranges, indicating the significant role of other factors, particularly vascular parameters, in the pathogenesis of glaucomatous damages [7]. Previous studies suggest that in these cases, the involvement of vascular factors, such as compromised blood flow to the optic nerve, is crucial in understanding and managing glaucoma beyond only the IOP levels [4,8-10]. So, vascular mechanisms in the pathogenesis of POAG have been an active area for research, and multiple studies have shown a link between glaucoma and alterations in intraocular pressure, systemic blood pressure, vasospasm, and disorders involving vascular alterations, like migraine and diabetes [11-17].

Fundus color stereoscopic photography studies revealed that retinal arterioles were significantly narrower in glaucoma patients compared to normal eyes, and while subsequent research confirmed consistent arteriole narrowing, venule changes were variable [4,18-21]. Doppler investigations further showed decreased flow velocity in the optic nerve head and indicated that autoregulation to reduced perfusion pressure contributes to higher IOP [13,22]. In patients with POAG, studies have shown an increase in diameter in major retinal vessels in the peripapillary area in response to a decrease in IOP, providing added evidence of vascular disease processes of glaucoma [23,24].

Prior research has been limited by the methods used to measure retinal vessel caliber, with a primary goal of reaffirming the role of vascular changes in POAG pathophysiology. This study aims to determine the correlation between retinal vessel caliber and visual field defects by measuring peripapillary major retinal vessels in eyes with primary open-angle glaucoma and established visual field defects, focusing on the superior, inferior, or both hemifields.

## Materials and methods

Study design

A cross-sectional study was conducted at a tertiary eye care center in northern Karnataka (Shri BM Patil Medical College, Hospital and Research Centre, Vijayapura) over one year, including 100 eyes from 50 patients diagnosed with POAG in both eyes. Written informed consent was obtained from all participants after a thorough briefing on the investigative procedures. Ethical clearance was provided by the Institutional Ethics Committee, Bijapur Lingayat District Educational University (BLDE) (Deemed to be University), Vijayapura, Karnataka, with approval number BLDE (DU)/IEC/992/2022-23; dated June 23, 2023. Patients were included in the study if they had a confirmed diagnosis of POAG including cases with normal-tension glaucoma, characterised by visual field defects in one or both hemifields as determined by standard automated perimetry, gonioscopically confirmed open anterior chamber angle, and characteristic optic disc changes such as increased cup-to-disc ratio, neuroretinal rim notching or thinning, or optic disc pallor. These patients were included regardless of their treatment status, whether they were undergoing medical management, had previously undergone surgical intervention, or were treatment-naive at the time of enrollment. Patients with secondary glaucoma, such as pigmentary glaucoma, pseudoexfoliative glaucoma, or uveitic glaucoma, were excluded to avoid confounding factors related to different pathophysiological mechanisms. Additionally, those with retinal vascular diseases like diabetic retinopathy or vascular occlusive diseases were excluded, as these conditions could independently affect the visual field and confound the assessment of glaucomatous damage. We also excluded patients with systemic hypertension due to the potential impact on vascular caliber and those with any history of trauma to the eye, which could lead to secondary optic nerve damage. Individuals with amblyopia, retinal pathologies like macular degeneration, dense cataracts, or a history of migraines were excluded to avoid confounding visual field assessment and optic disc evaluation.

Patient evaluation and data collection

All patients were evaluated by a physician to rule out hypertension, migraine or any other systemic vascular diseases. A comprehensive patient evaluation was conducted at baseline, including a detailed history. Clinical evaluation encompassed determining the best-corrected visual acuity with refractive error correction for accurate perimetry, slit lamp examination (Model: AIA-11-5S-L; Appasamy Associates, Chennai, India) of the anterior segment to detect underlying diseases, and intraocular pressure measurement with a Goldmann applanation tonometer (Model: AATM 5001; Appasamy Associates, Chennai, India) before pupil dilation for gonioscopy. Gonioscopy evaluation was performed using a four-mirror lens (Model: MIPL/14; Opticlear Ophthalmic Lenses, New Delhi, India), with Shaffer’s grading system used to grade the angles.

Visual field evaluation was carried out using a Humphrey automated visual field analyzer (Model: 740i; Zeiss) with a SITA (Swedish Interactive Threshold Algorithm) standard 30-2 test program and a size III stimulus on a white 31.5 apostlib background illumination. Total and pattern deviation plots and global indices were derived using the statistical software STATPAC (Carl Zeiss Meditec AG, Jena, Germany) for SITA version A10.1. Visual fields with unreliable indices were excluded and retested later. Minimal criteria for glaucomatous field defects were based on Anderson’s criteria, including “a glaucoma hemifield test outside normal limits, a pattern standard deviation with a p-value < 5%, and a cluster of three or more non-edge points on the pattern deviation plot in a single hemifield with p-values < 5%, one of which had a p-value < 1%” [25].

Optic disc evaluation was performed after achieving mydriasis with 0.8% tropicamide and 5% phenylephrine, followed by examination with a 90-diopter lens (Model: V90C; Volk, Mentor, OH, USA) for a stereoscopic view of the optic nerve head. Fundus photographs were captured using a retinal digital camera (Model: CX 1, Canon, Amsterdam, Netherlands), ensuring high quality and precise focus on retinal vessels. Measurements of the caliber of the superotemporal artery (STA), superotemporal vein (STV), inferotemporal artery (ITA), and inferotemporal vein (ITV) were taken manually by the same observer, who was blinded to the visual field defects. A concentric ring was placed over the optic disc in each image to facilitate the manual measurement of vessel calibers. The calibration factor, adjusted for camera optics and image resolution, was determined by measuring the distance from the macula to the optic disc in 20 photographs. The scale factor was calculated using the formula: Scale factor = 4500 micrometers/number of pixels, resulting in a calibration factor of 7.73 micrometers per pixel.

The severity of glaucoma was graded by categorizing patients into mild, moderate, severe, and end-stage glaucomatous damage. Mild glaucoma was defined by the presence of a nasal step or paracentral scotoma with a mean deviation (MD) of less than -6 dB. Moderate glaucoma was characterized by an arcuate scotoma with an MD between -6 dB and -12 dB. Severe glaucoma involved extensive field loss, including defects within the central 10 degrees, with an MD greater than -12 dB. End-stage glaucoma cases were defined as those in which visual field testing could not be performed due to the presence of a central scotoma [25]. The normal values for the STA: ITA ratio and STV: ITV ratio were calculated to be 0.954 and 0.942, referring to the study by Jonas et al. [26].

Statistical analysis

Statistical analysis was performed using the statistical software IBM SPSS Statistics for Windows, version 28 (IBM Corp., Armonk, NY, USA). The mean ratio of STA: ITA caliber and the STV: ITV caliber in eyes with superior hemifield visual field defects, inferior hemifield visual field defects, and both hemifield defects were compared using ANOVA (analysis of variance) and Scheffe multiple comparison test analysis. The number of eyes with ratios larger or smaller than the normal value in eyes with superior field defects, inferior field defects, and deficiencies in both hemifields was compared using chi-square analysis. A p-value of less than or equal to 0.05 is considered significant.

## Results

In this study, the mean age of participants was 89.68 years (± 11.17), with a gender distribution of 72% male (n=36) and 28% female (n=14). The mean best-corrected visual acuity (BCVA) in Log MAR (Logarithm of the Minimum Angle of Resolution) was 0.61 (± 0.34), and the mean intraocular pressure (IOP) was 20.01 mm Hg (± 7.71). Sixty percent of eyes (60 eyes) were on topical antiglaucoma medications, and 2% (two eyes) had undergone trabeculectomy. The distribution of visual field defects showed 20% (10 eyes) with superior arcuate scotoma, 32% (32 eyes) with inferior arcuate scotoma, 30% (30 eyes) with double arcuate scotoma, and 18% (18 eyes) with tubular vision. Among 100 eyes, 24 eyes were diagnosed with normal tension glaucoma. Regarding the grades of glaucoma, 52% of eyes (52 eyes) had moderate glaucoma, 30% (30 eyes) had severe glaucoma, and 18% (18 eyes) had end-stage glaucoma, with no eyes presenting mild glaucoma (Table 1).

**Table 1:** Baseline characteristics. SD: Standard deviation, BCVA: Best Corrected Visual Acuity, LogMAR: Logarithm of the Minimum Angle of Resolution, IOP: Intraocular pressure.

Baseline characteristics	Values
Mean age in years (± SD)	89.68 (± 11.17)
Gender*n (%)*	
-Male	36 (72%)
-Female	14 (28%)
Mean BCVA in Log MAR (± SD)	0.61 (± 0.34)
Mean IOP in mm Hg (± SD)	20.01 (± 7.71)
Patients on topical antiglaucoma medications*number of eyes (%)*	60 (60%)
Patients underwent trabeculectomy*number of eyes (%)*	2 (2%)
Distribution of field defects*number of eyes (%)*	
-Superior arcuate scotoma	20 (20%)
-Inferior arcuate scotoma	32 (32%)
-Double arcuate scotoma	30 (30%)
-Tubular vision	18 (18%)
Distribution of grades of glaucoma *number of eyes (%)*	
-Mild	0
-Moderate	52 (52%)
-Severe	30 (30%)
-End-stage	18 (18%)

**Figure 1:**
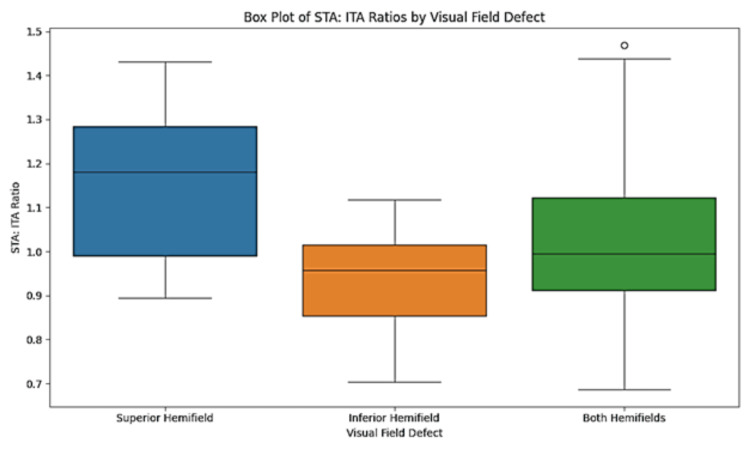
Box whisker plot showing the distribution of STA: ITA caliber ratio by visual field defects. STA: Superotemporal artery, ITA: Inferotemporal artery.

For superior hemifield defects (n=20), the mean STA: ITA ratio was 1.15 (± 0.18), with a p-value of 0.001, indicating statistical significance. Inferior hemifield defects (n=32) showed a mean STA: ITA ratio of 0.94 (± 0.12), with a p-value of 0.04, demonstrating statistical significance. When both hemifields were affected (n=48), the mean STA: ITA ratio was 1.04 (± 0.17), with a p-value of 0.04 (Table 2, Figure 1).

**Table 2:** Association between location of visual field defects with the ratio of superotemporal artery and inferotemporal artery calibers (STA: ITA ratio). STA: Superotemporal artery, ITA: Inferotemporal artery. P value less than 0.05 is considered statistically significant. ***: Very strongly significant, **: Strongly significant, *: Significant.

Visual field defects	Mean STA: ITA ratio	Standard deviation	P value
Superior hemifield (n=20)	1.15	0.18	0.001^***^
Inferior hemifield (n=32)	0.94	0.12	0.04^*^
Both hemifields (n=48)	1.04	0.17	0.04*

**Figure 2:**
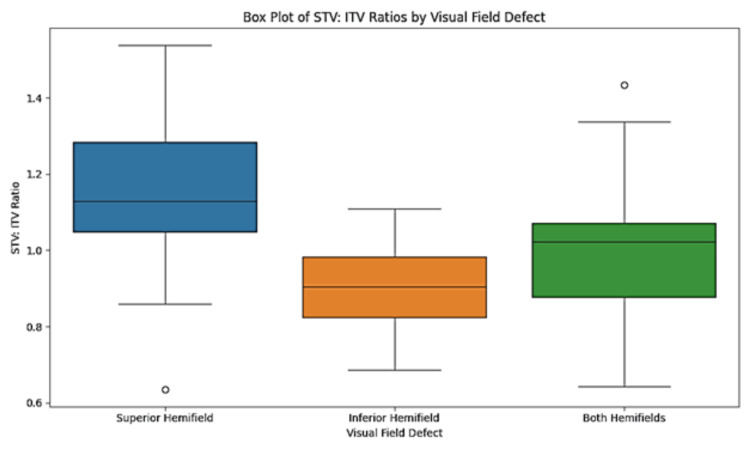
Box whisker plot showing the distribution of STV: ITV caliber ratio by visual field defects. STV: Superotemporal vein, ITV: Inferotemporal vein.

In cases with superior hemifield defects (n=20), the mean STV: ITV ratio was 1.13 (± 0.18), which was statistically significant with a p-value of <0.001. For inferior hemifield defects (n=32), the mean STV: ITV ratio was 0.90 (± 0.12), also demonstrating statistical significance with a p-value of <0.001. When both hemifields were affected (n=48), the mean STV: ITV ratio was 1.01 (± 0.17), with a p-value of 0.008 (Table 3, Figure 2).

**Table 3:** Association between the location of visual field defects with the ratio of superotemporal vein and inferotemporal vein calibers (STV: ITV ratio). STV: Superotemporal vein, ITV: Inferotemporal vein. P value less than 0.05 is considered statistically significant. ***: Very strongly significant, **: Strongly significant, *: Significant.

Visual field defects	Mean STV: ITV ratio	Standard deviation	P value
Superior hemifield (n=20)	1.13	0.18	< 0.001^***^
Inferior hemifield (n=32)	0.90	0.12	< 0.001^***^
Both hemifields (n=48)	1.01	0.17	0.008^**^

**Figure 3:**
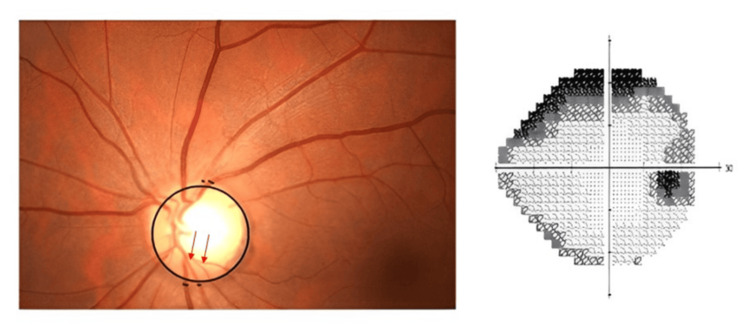
Fundus photograph showing 0.6 vertical cup disc ratio with inferior neuroretinal rim thinning (red arrow) in a patient with visual field analysis showing superior arcuate scotoma with narrowing of the inferotemporal artery and inferotemporal vein more than the superotemporal artery and vein.

Among 20 eyes with superior hemifield defects, 18 exhibited a ratio greater than the normal range. In the group of 32 eyes with inferior hemifield defects, an equal number of eyes had ratios either above or below the normal range. For the 48 eyes with defects in both hemifields, 36 showed ratios exceeding the normal range. Chi-square analysis revealed a significant p-value of 0.005 (Table 4, Figure 3).

**Table 4:** Comparison of STA: ITA caliber ratio less than and greater than normal by visual field defect location. STA: Superotemporal artery, ITA: Inferotemporal artery. P value less than 0.05 is considered statistically significant. ***: Very strongly significant, **: Strongly significant, *: Significant.

Visual field defect	STA: ITA ratios less than 0.954	STA: ITA ratios greater than 0.954	P value
Superior hemifield (n=20)	2	18	0.005^**^
Inferior hemifield (n=32)	16	16	
Both hemifields (n=48)	12	36	

**Figure 4:**
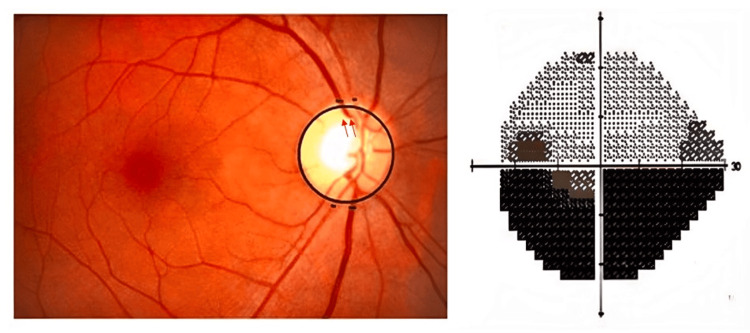
Fundus photograph showing 0.6 vertical cup disc ratio with superior neuroretinal rim thinning and notching (red arrow) in a patient with visual field analysis showing inferior arcuate scotoma with narrowing of the superotemporal artery more than the inferotemporal artery.

Among the 20 eyes with superior hemifield defects, all exhibited a ratio greater than the normal range. In the group of 32 eyes with inferior hemifield defects, 11 had ratios above the normal range. For the 48 eyes with defects in both hemifields, 30 showed ratios exceeding the normal range. Chi-square analysis indicated a highly significant result with p < 0.001 (Table 5, Figure 4).

**Table 5:** Comparison of STV: ITV caliber ratio less than and greater than normal by visual field defect location. STV: Superotemporal vein, ITV: Inferotemporal vein. P value less than 0.05 is considered statistically significant. ***: Very strongly significant, **: Strongly significant, *: Significant.

Visual field defect	STV: ITV ratios less than 0.942	STV: ITV ratios greater than 0.942	P value
Superior hemifield (n=20)	0	20	< 0.001^***^
Inferior hemifield (n=32)	21	11	
Both hemifields (n=48)	18	30	

## Discussion

Several research studies have provided evidence in favor of the vascular theory of glaucoma, which links the disease to changes in blood vessels [16,23,27,28]. Studies indicate that non-glaucomatous optic atrophy also exhibits retinal artery narrowing, but parapapillary chorioretinal atrophy assessment can help distinguish between non-glaucomatous and glaucomatous optic neuropathy [29-31]. In this study, we assessed peripapillary retinal vessel diameters and calculated their mean ratios using fundus photographs and a validated calibration factor. These parameters were then correlated with visual field defects observed in one or both hemifields. Visual field defects included superior arcuate scotoma (20%), inferior arcuate scotoma (32%), double arcuate scotoma (30%), and tubular vision (18%). Glaucoma severity was moderate in 52% of eyes, severe in 30%, and end-stage in 18%. The mean STA: ITA ratio was significantly higher for superior hemifield defects (p = 0.001) and lower for inferior hemifield defects (p = 0.04). Similarly, STV: ITV ratios were significantly altered for both hemifield defects and were statistically significant.

The mean age of the participants in this study was 89.68 years (± 11.17), with a male preponderance of 72%. A study conducted in similar demography by Mudhol and Ray reported a mean age of 66.93 years (±10.06), which is on the lower side compared to our study [32]. The higher average age in our study may be attributed to the inclusion of bilateral open-angle glaucoma, a condition that progresses gradually and often takes time to affect both eyes. They reported a male preponderance of 63%, which aligns with the current study [32]. The mean intraocular pressure (IOP) in our study was 20.01 mm Hg (± 7.71), which is lower than the 36.1 mm Hg (± 8.50) reported by Kortuem et al. [9]. The lower IOP observed in our study can be attributed to the inclusion of normal tension glaucoma among the POAG.

Our findings demonstrate a statistically significant narrowing of the STA in eyes with inferior hemifield defects and a narrowing of the ITA in eyes with superior hemifield defects. This aligns with Jonas et al., who identified a correlation between the caliber of parapapillary vessels and the presence of glaucomatous damage [26]. Furthermore, significant changes in STV: ITV caliber corresponding to respective hemifield defects were also observed, corroborating previous findings that glaucomatous damage affects both retinal arteries and veins [28,33].

Comparative analysis of STA: ITA ratios showed that 90% of eyes with superior hemifield defects had ratios above normal, indicating a significant narrowing of the ITA. In eyes with inferior hemifield defects, 50% had ratios above the normal range, and 50% had ratios below the normal range. For the eyes with defects in both hemifields, 75% had ratios above the normal range. These findings reflect the differential impact of glaucomatous damage on retinal vascular anatomy and are consistent with Pakter et al., who found that variations in retinal vascular caliber are linked to different patterns of visual field loss in POAG [34]. In cases where both hemifields were affected, there was a notable predominance of increased ratios, particularly in the inferior vessels. This suggests bilateral visual field defects are associated with more severe vascular changes, especially in the inferior peripapillary region. This result corroborates earlier studies indicating that glaucomatous damage often manifests more prominently in the inferotemporal region of the optic disc [35].

Previous research on the impact of antiglaucoma medications on ocular blood flow has produced mixed results, with some studies suggesting beneficial effects while others report minimal impact [36,37]. In the current cross-sectional study, 60% of the eyes were receiving antiglaucoma medications, which may influence vascular caliber. Further follow-up is necessary to accurately assess the impact of these medications on vascular changes.

This study effectively evaluates retinal vessel calibers and their correlation with visual field defects in POAG using precise imaging techniques and a well-defined calibration factor. The analysis of a diverse patient cohort adds depth to the findings. However, the cross-sectional design limits causal inference, and the study’s focus on a single tertiary center with a smaller sample size may affect generalizability. Exclusion of secondary glaucomas and comorbidities may limit applicability to a broader patient population. Future studies using Optical Coherence Tomography Angiography (OCTA) could offer additional insights into microvascular changes, potentially complementing our findings on peripapillary vessel caliber in POAG.

## Conclusions

There is a significant correlation between the narrowing of the inferior retinal vascular caliber and abnormalities in the superior visual field in POAG, and vice versa. Notably, eyes with defects in both hemifields show a pronounced reduction in the caliber of inferotemporal retinal vessels. These vascular patterns suggest that the extent of vessel narrowing is associated with the degree of corresponding visual field loss, making it a potentially valuable diagnostic tool for assessing glaucomatous damage. However, this finding needs further validation through studies with larger sample sizes, longer follow-up periods, and more diverse populations.
